# DUET-Net: A Deformable CNN–Transformer with Calibrated Dual Uncertainty for Brain Tumor MRI Classification

**DOI:** 10.3390/diagnostics16213624

**Published:** 2026-11-02

**Authors:** Md Ariful Islam Mozumder, Proloy Kumar Mondal, Hee Cheol Kim, Suliman Aladhadh

**Affiliations:** 1 Institute of Digital Anti-Aging Healthcare, Inje University, Gimhae-si 50834, Republic of Korea; 2 Department of Information Technology, College of Computer, Qassim University, Buraydah 52571, Saudi Arabia

**Keywords:** brain tumor classification, MRI, CNN, vision transformer, attention mechanism, deep learning, explainable AI

## Abstract

**Background:** Magnetic resonance imaging (MRI) is a primary modality for brain tumor diagnosis and treatment planning, making accurate automated classification critically important. However, existing deep learning approaches face limitations in prediction reliability, generalization across imaging domains, and calibrated confidence estimation. To address these gaps, we introduce DUET-Net, a hybrid CNN–Transformer framework that integrates six complementary architectural innovations. **Methods:** First, a multiscale depthwise fusion (MSDF) module aggregates multi-receptive-field context from a single 2D MRI slice using parallel depthwise convolutions at three kernel sizes. Second, domain-adaptive batch normalization (DABN) with input-conditioned mixture weights maintains domain-specific statistics while sharing affine parameters, enabling sample-adaptive generalization across imaging sources. Third, an enhanced convolutional block attention module (CBAM) introduces deformable spatial offsets to adaptively attend to irregular tumor borders. Fourth, a deformable cross-scale attention (DCSA) module combines per-scale learnable spatial offsets with adaptive cross-scale weighting for geometry-aware multi-resolution fusion. Fifth, a hierarchical Vision Transformer encoder coupled with Monte Carlo dropout and a heteroscedastic aleatoric head enables dual uncertainty estimation grounded in the Kendall–Gal framework. Sixth, post-hoc temperature calibration and Grad-CAM++ provide trustworthy confidence scores and visual interpretability. **Results:** The proposed model achieves 99.51% accuracy on the Kaggle dataset, 95.27% on the Figshare dataset, and 99.89% on the Mendeley dataset with an expected calibration error (ECE) of 0.0142. With approximately 48 M parameters and 6.66 GFLOPs, DUET-Net sustains 52.41 FPS and is the only compared method to provide calibrated uncertainty estimation. Ablation, cross-dataset, and uncertainty-formulation comparison studies further validate the reliability of the framework. **Conclusions:** The potential of our proposed net as a promising approach for reliable computer-assisted brain tumor classification, although further external and prospective clinical validation is necessary before deployment.

## 1. Introduction

Brain tumors remain among the most challenging neurological malignancies worldwide because of their aggressive progression, high morbidity, and substantial mortality burden. According to the latest estimates from the World Health Organization (WHO) and the International Agency for Research on Cancer (IARC), approximately 321,731 new cases of brain and central nervous system (CNS) tumors and 248,500 related deaths occurred globally in 2022, highlighting the significant public health impact of these diseases [1]. The incidence rate for 2022 in Western Europe was 5.56 per 100,000 people, which is almost as high as the rate for North America (5.46), while East Asia (3.95) and Africa (2.1) had comparatively lower incidences [2]. The regional differences highlight the worldwide impact of brain tumors and the differences in access to diagnostic facilities across health systems. Brain tumors are formed by abnormal multiplication of cells and can be either benign or malignant, with malignant tumors having fast growth rates and the ability to spread into other tissues of the brain [3,4]. Symptoms range from chronic headache, seizures, vision and speech changes, to cognitive impairments [5]. Hence, early and precise classification is of pivotal importance as it directly affects the selection of treatment, surgical planning, and hence the prognosis of the patient [6].

Magnetic resonance imaging (MRI) has become the standard imaging method for visualizing brain tumors because of its higher soft-tissue contrast than computed tomography (CT) [7]. While MRI has many advantages, it also has a number of drawbacks during diagnosis. Specifically, morphological heterogeneity within the same histological subtype leads to significant intra-class variability, while the visual similarity among different tumor types leads to inter-class confusion [8]. There are also difficulties in manual interpretation by radiologists due to inter-observer variation, fatigue, and the difficulty of precisely defining tumor boundaries in complex imaging data [9]. Such restrictions have inspired the design of computer-aided diagnostic (CAD) systems using artificial intelligence to increase accuracy and consistency. 

Brain tumor classification has undergone various stages of methodology. Initial models were based on handcrafted feature extraction techniques, such as gray-level cooccurrence matrices (GLCM), discrete wavelet transforms (DWT), and histogram of oriented gradients (HOG), and on classic machine learning classifiers, including support vector machines (SVM), k-nearest neighbors (k-NN), and random forests. While moderate accuracy was achieved with those approaches, they were time-consuming, required significant domain knowledge, and were not easily scalable. The emergence of deep learning, especially convolutional neural networks (CNNs), fundamentally changed the discipline because it allowed automatic learning of hierarchical features using only pixel data in the form of images or videos directly through deep learning algorithms. Transfer learning has also improved medical imaging tasks lacking data [10,11].

Most recently, Vision Transformer (ViT) [12] has been shown to have high capacity to capture long-range dependencies. However, pure transformer architectures require large amounts of data and are not easily used to perform fine-grained local feature extraction. This has resulted in hybrid CNN-Transformer networks which incorporates local feature learning and global context modelling [13,14].

Despite these advances, existing methods suffer from several key limitations that hinder clinical deployment:

- Absence of Uncertainty Quantification: Most models provide deterministic predictions without reliability estimation, limiting clinical trust.
- Limited Cross-Domain Generalization: Models often fail to generalize across different scanners, protocols, and populations due to domain shift.
- Insufficient Multi-Scale Feature Integration: Existing approaches inadequately capture tumor characteristics across multiple spatial scales.
- Lack of Calibrated Confidence: Predicted probabilities are often miscalibrated, leading to unreliable confidence estimates.

To address these challenges, we propose DUET-Net, a hybrid CNN–Transformer framework with the following key contributions:

- Multi-Scale Depthwise Fusion (MSDF): Parallel depthwise convolutions with receptive fields of 3 × 3, 5 × 5, and 7 × 7 capture multi-scale contextual information from a single 2D MRI slice.
- Domain-Adaptive Batch Normalization: Maintains domain-specific statistics for improved generalization.
- Enhanced CBAM: Incorporates deformable spatial attention for irregular tumor boundaries.
- Deformable Cross-Scale Attention (DCSA): Combines per-scale learnable spatial offsets with adaptive cross-scale weighting for geometry-aware multiresolution fusion.
- Dual Uncertainty Framework: Combines Monte Carlo dropout and aleatoric modeling for reliable predictions.
- Calibrated Confidence and Explainability: Integrates temperature scaling and Grad-CAM++ for clinical interpretability.

We evaluate DUET-Net on three publicly available MRI datasets, namely Kaggle, Figshare, and Mendeley, demonstrating state-of-the-art classification performance, strong calibration (ECE = 0.0142), and efficient computational characteristics (approximately 48 M parameters, 6.66 GFLOPs, and 52.41 FPS). Comprehensive ablation studies, cross-dataset generalization experiments, and uncertainty formulation comparisons further validate the effectiveness, robustness, and clinical applicability of the proposed framework.

The remainder of this paper is organized as follows. Section 2 reviews related work. Section 3 details the proposed DUET-Net architecture, along with datasets and implementation. Section 4 presents the experimental results, including ablation, uncertainty, and cross-dataset analyses. Section 5 discusses the findings, Section 6 presents the limitations of the study, and Section 7 concludes the paper.

## 2. Related Work

Brain tumor classification using magnetic resonance imaging (MRI) has been well investigated, evolving from traditional machine learning methods to deep learning and hybrid methods that integrate attention mechanisms and vision transformers. The initial approaches were based on handmade feature extraction and standard classifiers. A variety of techniques were employed in conjunction with classifiers such as support vector machines (SVMs), k-nearest neighbors (k-NN), and random forests, including gray-level co-occurrence matrices (GLCMs), discrete wavelet transforms (DWTs) and the histogram of oriented gradients (HOGs). For example, Zacharaki et al. [15] used SVM and k-NN to classify glioma grades, and El-Dahshan et al. [16] used DWT-PCA to obtain good binary classification accuracy. While these approaches laid the groundwork for future methodologies, they relied on handcrafted features, were not scalable, and failed to capture complex tumor characteristics.

Deep learning, in particular convolutional neural networks (CNNs), has enabled a huge leap forward in brain tumor classification by introducing automatic hierarchical feature learning. The CNN-based approaches proposed by Paul et al. [17] and Anaraki et al. [18] showed better performance than classical methods. Transfer learning has also improved performance in medical imaging applications with limited data, achieving accuracies of more than 96% using models such as VGG19, DenseNet, and EfficientNet [10,11]. To enhance robustness, hybrid CNN-based models combined with optimization methods or classifiers, such as SVM, have also been investigated. However, despite their high accuracy, these models generally lack uncertainty estimation, calibrated confidence, and strong cross-domain generalization.

In CNN architectures, attention mechanisms have been introduced to address challenges in feature representation and to improve discriminative learning capability. Attention-based CNN models focus on informative regions and achieve better classification performance. Furthermore, the integration of CNNs with Vision Transformers (ViTs) has advanced the field by combining local feature extraction with global contextual relationship modeling. Transformer-based approaches such as RViT [19] and hybrid CNN–Transformer models have demonstrated promising effectiveness.

Uncertainty quantification has become a key need for clinical deployment but has yet to be fully investigated for brain tumor classification. For instance, Monte Carlo dropout [20] is a method of approximating Bayesian inference through stochastic forward pass during the inference stage. Some methods have been suggested to model epistemic and aleatoric uncertainties, such as deep ensemble methods or heteroscedastic modeling. Although these methods have been used in segmentation and other medical imaging applications, they have not been widely used in brain tumor classification systems. Current methods generally offer only deterministic predictions, but without quantifying the reliability of the prediction.

A second important issue is domain shift, i.e., the phenomenon that models trained on one dataset do not perform well on different imaging protocols or even different institutions. The use of domain adaptation methods, especially batch normalization, has been found to yield positive results. Models can adapt to data distribution variations using adaptive batch normalization (AdaBN) and domain-specific normalization strategies. In addition, how to improve representation robustness has been studied using contrastive learning, which aims to promote intra-class compactness and inter-class separation. The integration of domain adaptation with contrastive learning and uncertainty estimation in a single model has not been extensively studied for brain tumor classification.

Deformable convolutions and multi-scale feature fusion techniques have been developed recently, and these methods have successfully enhanced model flexibility to better handle complex tumor structures. Deformable convolutional networks adjust the sampling points in the space to better represent irregular shapes, while multi-scale feature fusion techniques fuse information from multiple scales. While promising approaches for object detection and segmentation, these techniques are not yet widely incorporated into hybrid CNN–Transformer architectures for brain tumor classification.

In Table 1, we compare the existing approaches in terms of six aspects important for clinical deployment. The motivation behind the development of DUET-Net is to solve all six dimensions in one end-to-end trainable framework: (i) deformable multi-scale attention to handle tumor morphological heterogeneity, (ii) dual uncertainty quantification for risk-aware predictions, (iii) calibrated confidence scores for reliable decision support, (iv) cross-domain generalization for multi-site deployment, (v) efficient inference for real-time clinical use, and (vi) interpretable visual explanations.

As shown in Table 1, DUET-Net is the only framework that satisfies all six clinical deployment criteria. This comprehensive integration of deformable multi-scale attention, dual uncertainty quantification, calibrated confidence, domain-adaptive normalization, computational efficiency, and visual explainability represents the principal contribution of this work and distinguishes it from all existing approaches in the literature.

## 3. Proposed Method

### 3.1. Overall Architecture

The proposed DUET-Net architecture is designed as a comprehensive hybrid framework that addresses four critical gaps in existing brain tumor classification methods: (i) the lack of volumetric context in slice-level analysis, (ii) insufficient cross-scale feature interaction, (iii) the absence of prediction uncertainty quantification, and (iv) limited cross-domain generalization capability. The architecture comprises six novel components integrated into an end-to-end trainable pipeline: a multi-scale depthwise fusion (MSDF) module, a ResNet-50 backbone with domain-adaptive batch normalization, enhanced CBAM with deformable spatial attention, a deformable cross-scale attention (DCSA) module, an uncertainty-aware hierarchical transformer encoder, and a dual-head classification module with calibrated confidence scoring.

**Figure 1:**
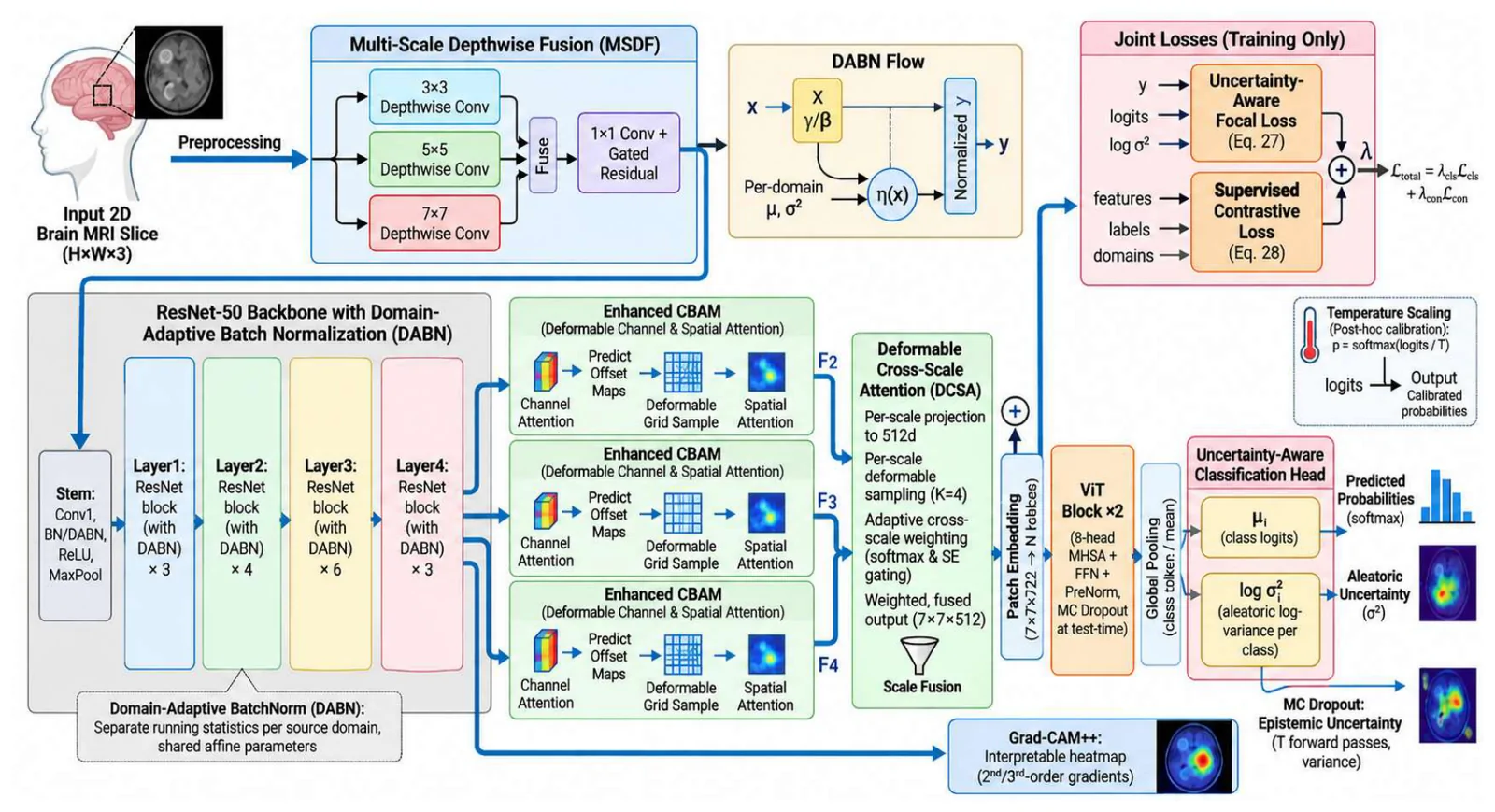
Overview of the proposed DUET-Net architecture.

Given an input MRI image ${X\in R}^{H\times W\times 3}$ with spatial dimensions *H* = *W* = 224, the network produces a probability distribution over C tumor classes along with per-sample epistemic and aleatoric uncertainty estimates. The schematic view of the entire proposed model is shown in Figure 1. The complete architectural configuration of DUET-Net, including the output shapes and design specifications of each component, is summarized in Table 2.

### 3.2. Multi-Scale Depthwise Fusion (MSDF) Module

To enrich the input representation with multi-receptive field context while keeping computational cost low, we introduce a multi-scale depthwise fusion (MSDF) module at the network input. Unlike volumetric 2.5D or 3D approaches that require adjacent MRI slices—which are not consistently available across our three benchmark datasets—MSDF operates on a single 2D slice and aggregates spatial context at multiple receptive fields using parallel depthwise separable convolutions. This design provides part of the contextual benefit traditionally associated with 2.5D processing (varying receptive-field aggregation) without requiring volumetric inputs; a genuine 2.5D extension is discussed in Section 6. Three parallel branches process the input at kernel sizes 3 × 3, 5 × 5, and 7 × 7:

(1) $$B_{k}\left(X\right)=ReLU\left(BN\;\left({DWConv}_{k\times k}\left(X\right)\right)\right),\;k\in \{3,5,7\}$$

where DWConv denotes depthwise convolution, and BN is batch normalization. The multi-scale features are concatenated and fused through a pointwise convolution:

(2) $$F_{fused}=ReLU\left(BN\left({Conv}_{1\times 1}\left(\left[B_{3};B_{5};B_{7}\right]\right)\right)\right)$$

A learnable gating mechanism controls the residual connection:

(3) $$\alpha =\sigma \left(W_{g}\cdot GAP\left(F_{fused}\right)\right)$$

(4) $$\hat{X}=X+\alpha ⊙F_{fused}$$

where GAP denotes global average pooling and *σ* is the sigmoid function. This gated residual design ensures that the original signal is preserved while selectively incorporating multi-scale contextual information.

### 3.3. Domain-Adaptive Batch Normalization

To enhance cross-dataset generalization, we replace standard batch normalization layers with domain-adaptive batch normalization (DABN). DABN maintains separate running statistics ($\mu_{d}$, $\sigma_{d}^{2}$) for each of *D* source domains while sharing the learnable affine parameters *γ* and *β* across all domains. During training on a batch from domain *d*, only the statistics of domain *d* are updated (Equation (5)). At inference, we depart from prior AdaBN-style methods that use fixed mixture weights and instead compute input-conditioned domain weights via a lightweight gating MLP $g_{\varphi }$:

(5) $$\hat{x}=\gamma \cdot \frac{x-\mu_{d}}{\sqrt{\sigma_{d}^{2}+\epsilon }}+\beta$$

During inference, a learned soft mixture of domain statistics is employed:

(6a) $$\pi \left(x\right)=softmax\left(g_{\phi }\left(GAP\left(x\right)\right)\right)\in R^{D}$$

(6b) $$\mu_{mix}\left(x\right)=\sum_{d}\pi_{d}\left(x\right)\mu_{d},\;\sigma_{mix}^{2}\left(x\right)=\sum_{d}\pi_{d}\left(x\right)\sigma_{d}^{2}$$

where $\pi_{d}$ = softmax ($w_{d}$) are learnable domain mixing weights. This formulation enables the model to adapt its normalization behavior to unseen domains without requiring domain labels at test time.

Here, *gϕ* is a two-layer MLP (*C* → *C*∕4 → *D*) with GELU activation and approximately 0.05 M parameters per layer. This input-conditioned formulation allows each test sample to receive a personalized convex combination of source statistics, providing adaptation to unseen scanners and acquisition protocols without requiring domain labels at test time. When trained on a single source (*D* = 1), DABN reduces exactly to standard batch normalization (BN); the multi-source benefit is evaluated explicitly in DABN isolation study.

### 3.4. CNN Backbone with Enhanced CBAM

We employ ResNet-50 pre-trained on ImageNet as the backbone, decomposed into a stem module and four residual stages (Layer1–Layer4). The feature hierarchy is expressed as follows:

(7) $$F_{l}={Layer}_{l}\left(F_{l-1}\right),\;l\in \{1,2,3,4\}$$

where $F_{1}$ ∈ ℝ 256 × 56 × 56, $F_{2}\;$∈ ℝ 512 × 28 × 28, $F_{3}\;$∈ ℝ 1024 × 14 × 14, and $F_{4}$ ∈ ℝ 2048 × 7 × 7. Enhanced CBAM modules are integrated after Layer2, Layer3, and Layer4.

#### 3.4.1. Channel Attention

The channel attention map is computed as in the original CBAM:

(8) $$M_{c}\left(F\right)=\sigma \left(MLP\left(GAP\left(F\right)\right)+MLP\left(GMP\left(F\right)\right)\right)$$

#### 3.4.2. Deformable Spatial Attention

Unlike the original CBAM, which uses a fixed-kernel spatial attention, we introduce a deformable spatial attention mechanism that learns input-dependent sampling offsets. Given the channel-refined feature **F**′:

(9) $$\Delta p=s\cdot tanh\left({Conv}_{3\times 3}\left({Conv}_{3\times 3}\left(F\prime \right)\right)\right)$$

where *s* is a learnable scale parameter initialized to 0.1 and Δ**p** ∈ ℝ 2 × *H* × *W* represents the *x* and *y* offsets for each spatial location. The deformed feature is obtained via bilinear grid sampling:

(10) $$F_{def}=GridSample\left(F\prime ,G_{base}+\Delta p\right)$$

The spatial attention map is then applied to the deformed features:

(11) $$M_{s}\left(F_{def}\right)=\sigma \left({Conv}_{7\times 7}\left(\left[AvgPool\left(F_{def}\right);MaxPool\left(F_{def}\right)\right]\right)\right)$$

(12) $$F\prime \prime =M_{s}\left(F_{def}\right)⊙F_{def}$$

This deformable mechanism enables the attention to adaptively focus on irregular tumor boundaries rather than being constrained to fixed rectangular receptive fields, which is particularly beneficial for tumors with heterogeneous morphology.

### 3.5. Deformable Cross-Scale Attention (DCSA)

To exploit multi-scale representations while enabling geometry-aware spatial alignment across feature levels, we introduce DCSA, which combines (i) per-scale learnable spatial offsets and (ii) adaptive cross-scale weighting.

#### 3.5.1. Per-Scale Projection

Each backbone scale $F{\prime \prime }_{l}$ (*l* ∈ {2, 3, 4}) is first projected to a common dimension *d* = 512;

(13) $$P_{l}=ReLU\left(BN\left({Conv}_{1\times 1}\left(F{\prime \prime }_{l}\right)\right)\right),l\in \{2,3,4\}$$

#### 3.5.2. Deformable Sampling

A lightweight offset network predicts 2*K* sampling offsets per spatial location (with *K* = 4 sampling points):

(14) $$\Delta p_{l}=s_{l}\cdot tanh\left({Conv}_{3\times 3}\left(GELU\left({Conv}_{3\times 3}\left(P_{l}\right)\right)\right)\right)\in R^{2K\times H_{l}\times W_{l}}$$

(15) $$P_{l}^{def}\left(p\right)=\frac{1}{K}\sum_{k=1}^{K}GridSample\left(P_{l},p+\Delta p_{l}^{\left(k\right)}\right)$$

where $s_{l}$ is a per-scale learnable scalar initialized to 0.1 for training stability. This allows each scale to align its sampling grid to tumor-relevant geometry before fusion.

#### 3.5.3. Adaptive Cross-Scale Weighting

All deformed features are resized to the target spatial resolution (7 × 7) via bilinear interpolation, and cross-scale attention weights are computed from global descriptors:

(16) $$\omega =softmax\left(W_{s}\cdot \left[GAP\left(P_{2}^{def}\right);GAP\left(P_{3}^{def}\right);GAP\left(P_{4}^{def}\right)\right]\right)$$

#### 3.5.4. Fusion with Squeeze-Excitation Gating


(17) $$F_{fused}=SE\left(\sum_{l}\omega_{l}\cdot P_{l}^{def}\right)$$

The combination of per-scale deformable sampling and adaptive cross-scale weighting allows DCSA to both localize irregular tumor geometry and select the optimal resolution for each query.

### 3.6. Uncertainty-Aware Hierarchical Transformer Encoder

The fused multi-scale features are tokenized through a patch embedding layer using a 2 × 2 convolution with stride 2, producing *N* = 9 tokens of dimension *d* = 512. Learnable positional embeddings are added:

(18) $$Z_{0}=PatchEmbed\left(F_{fused}\right)+E_{pos}$$

Two sequential transformer blocks with pre-normalization process the tokens:

(19) $$Z\prime_{l}=MHSA\left(LN\left(Z_{l}\right)\right)+Z_{l}$$

(20) $$Z_{l+1}=FFN\left(LN\left(Z\prime_{l}\right)\right)+Z\prime_{l}$$

where MHSA employs ℎ = 8 attention heads with GELU activation in the FFN ($d_{ff}$ = 1024). Critically, dropout with rate *p* = 0.15 is applied within each transformer block and is kept active during inference to enable Monte Carlo (MC) dropout for epistemic uncertainty estimation.

Note that the dropout rate *p* = 0.15 within transformer blocks serves a dual purpose: regularization during training and Monte Carlo (MC) sampling during inference for epistemic uncertainty estimation (Equations (21) and (22)).

### 3.7. Dual-Head Classification with Calibrated Uncertainty

#### 3.7.1. Heteroscedastic Aleatoric Uncertainty Head

Following Kendall and Gal [28], we adopt a heteroscedastic noise model for classification. For each input $x_{i}$, after adaptive average pooling across the token sequence to obtain $z_{i}$, the network predicts class logits $\mu_{i}$ ∈ $R^{c}$ and an input-dependent log-variance $log\sigma_{i}^{2}$ ∈ $R^{c}$ representing observation noise:

(21) $$\mu_{i}=W_{\mu }\cdot z_{i}+b_{\mu }$$

(22) $$log\sigma_{i}^{2}=MLP\left(z_{i}\right)$$

The aleatoric uncertainty captures noise inherent in the data itself, such as ambiguous tumor boundaries, imaging artifacts, or partial volume effects at tumor edges. The Gaussian-corrupted softmax likelihood derivation yields the heteroscedastic loss formulation given in Equation (28), which we further extend with focal weighting in Section 3.8.1.

#### 3.7.2. Epistemic Uncertainty via MC Dropout

During inference, we perform $T_{MC}$ = 10 stochastic forward passes with dropout active (notation distinct from the calibration temperature $T_{cal}$ in Equation (24)):

(23) $$\bar{p}=\frac{1}{T_{MC}}\sum_{t=1}^{T_{MC}}softmax\left(\mu_{t}\right)$$

(24) $$\Sigma_{epi}=\frac{1}{T_{MC}}\sum_{t=1}^{T_{MC}}{\left(softmax\left(\mu_{t}\right)-\bar{p}\right)}^{2}$$

(25) $$\Sigma_{ale}=\frac{1}{T_{MC}}\sum_{t=1}^{T_{MC}}exp\left(log\sigma_{t}^{2}\right)$$

We empirically verified that increasing $T_{MC}$ to 30 and 50 yielded negligible improvement (mean epistemic uncertainty changed by less than $10^{-3}$ across all three datasets), supporting that $T_{MC}$ = 10 is sufficient for reliable uncertainty estimation while remaining computationally efficient. This dual uncertainty quantification enables clinicians to distinguish between cases where the model lacks knowledge (high epistemic uncertainty, potentially addressable with more training data) and cases where the input data are inherently ambiguous (high aleatoric uncertainty, requiring additional clinical examination).

#### 3.7.3. Temperature Calibration

Post-hoc temperature scaling is applied to ensure well-calibrated confidence scores. A scalar temperature $T_{cal}$ is learned on a held-out validation set by minimizing the negative log-likelihood:

$$p_{cal}=softmax\left(\frac{\mu }{T_{cal}}\right)$$

The expected calibration error (ECE) quantifies calibration quality:

(26) $$ECE=\sum_{m}\frac{n_{m}}{N}\left|acc\left(B_{m}\right)-conf\left(B_{m}\right)\right|$$

The temperature parameter is estimated using the validation data by minimizing the negative log-likelihood of the predicted class probabilities. The optimized temperature is subsequently applied to the model logits during inference to obtain calibrated confidence scores. The held-out test data are not used for temperature optimization.

#### 3.7.4. Grad-CAM Explainability

To provide interpretable visual explanations, we employ Grad-CAM++, which uses weighted combinations of positive partial derivatives of the last convolutional layer activations. Unlike standard Grad-CAM, which uses global average-pooled gradients, Grad-CAM++ computes pixel-wise weights:

(27) $$\alpha_{ij}^{kc}=\frac{\frac{\partial^{2}Y^{c}}{\partial {\left(A_{ij}^{k}\right)}^{2}}}{\frac{\partial^{2}Y^{c}}{\partial {\left(A_{ij}^{k}\right)}^{2}}+\sum_{a,b}A_{ab}^{k}\frac{\partial^{3}Y^{c}}{\partial {\left(A_{ij}^{k}\right)}^{3}}}$$

where $Y^{c}$ denotes the score for class *c*, $A_{ij}^{k}$ represents the activation of the *k*-th feature map at spatial location (*i*, *j*), and the summation over (*a*, *b*) spans all spatial locations of the feature map. The resulting heatmaps provide more precise localization of tumor regions compared to standard Grad-CAM.

#### 3.7.5. Relation to Alternative Uncertainty Formulations

Our choice of heteroscedastic noise modeling is one of several established paradigms for predictive uncertainty estimation. Evidential deep learning (EDL) [29] places a Dirichlet prior over class probabilities and outputs evidence parameters ***α***, providing closed-form aleatoric–epistemic decomposition without MC sampling. Logit-noise injection [30] models noise directly on pre-softmax activations and supports correlated label-noise structures. Bayesian neural networks with variational inference [31] offer fully probabilistic weight posteriors but at substantial computational cost.

We selected the heteroscedastic focal formulation for three pragmatic reasons:

1. Compositional simplicity: It composes naturally with MC dropout, providing both aleatoric and epistemic estimates without architectural duplication or auxiliary networks.
2. Parameter efficiency: It requires only a scalar log variance head per class (∼2 K parameters total in our implementation), compared to EDL’s full evidence vector which scales with class count and feature dimension.
3. Empirical calibration: It achieved the lowest expected calibration error in our preliminary experiments on DS-2, representing the most challenging dataset. A direct empirical comparison against EDL and other baselines is reported in Table 16.

### 3.8. Joint Loss Function

The total training objective combines two complementary loss terms:

(28) $$L_{total}=\lambda_{cls}L_{cls}+\lambda_{cont}L_{cont}$$

Note that aleatoric uncertainty regularization is implicitly incorporated within $L_{cls}$ through the $\frac{1}{2}log\sigma_{i}^{2}$ term in Equation (27) (the focal-heteroscedastic loss in Section 3.8.1), which automatically balances classification confidence with the model’s predicted uncertainty. This eliminates the need for a separate uncertainty loss weight.

#### 3.8.1. Uncertainty-Aware Focal Loss

Building on the heteroscedastic head in Section 3.7.1, we derive the classification loss in two steps. Following Kendall and Gal’s Monte Carlo negative log-likelihood (NLL) estimate for heteroscedastic classification [28]:

(29) $$L_{NLL}\approx \frac{1}{N}\sum_{i=1}^{N}\left[\frac{1}{2}exp\left(-log\sigma_{i}^{2}\right)\cdot CE\left(\mu_{i},y_{i}\right)+\frac{1}{2}log\sigma_{i}^{2}\right]$$

The first term down-weights cross-entropy contributions from samples with high predicted variance, while the second term regularizes $log\sigma_{i}^{2}$ and prevents it from diverging to +∞.

To additionally focus learning on hard examples—particularly important under the severe inter-class similarity observed in DS-2 between glioma and meningioma—we substitute focal cross-entropy [32] for the standard CE term in Equation (29), yielding our final heteroscedastic-focal classification loss:

(30) $$L_{cls}=\frac{1}{N}\sum_{i=1}^{N}\left[\frac{1}{2}exp\left(-log\sigma_{i}^{2}\right)\cdot {\left(1-p_{i}\right)}^{\gamma }\cdot {CE}_{i}+\frac{1}{2}log\sigma_{i}^{2}\right]$$

where *γ* = 2.0 is the focal parameter and *pi* is the predicted probability of the correct class. This formulation simultaneously (i) down-weights samples the model identifies as ambiguous via high $log\sigma_{i}^{2}$, preventing overconfidence, and (ii) emphasizes hard examples via focal weighting, addressing class imbalance and boundary-confusion cases.

#### 3.8.2. Supervised Contrastive Loss

To learn domain-invariant representations, features from the contrastive projection head are used to compute a supervised contrastive loss:

(31) $$L_{cont}=-\frac{1}{\left|P\left(i\right)\right|}\sum_{p\in P\left(i\right)}log\left[\frac{exp\left(z_{i}\cdot z_{p}/\tau \right)}{\sum_{a\neq i}exp\left(z_{i}\cdot z_{a}/\tau \right)}\right]$$

where *τ* = 0.07 is the temperature and P(*i*) denotes the set of positive pairs (same class) for anchor *i*. This loss encourages same-class samples to cluster together in the feature space regardless of their source domain.

### 3.9. Implementation Details

DUET-Net is implemented in PyTorch (2.12.0). The ResNet-50 backbone is initialized with ImageNet pre-trained weights. Input images are resized to 224 × 224 and normalized (mean = 0.5, std = 0.5). Data augmentation includes random horizontal flipping, rotation (±15°), affine translation (5%), color jittering (brightness/contrast ±10%), random grayscale (5%), and random erasing (10%). The model is trained using the AdamW optimizer with learning rate 1 × $10^{-4}$ and weight decay 1 × $10^{-4}$. Cosine annealing with warm restarts ($T_{0}$ = 10, $T_{mult}$ = 2) is employed. Gradient clipping at max norm 1.0 is applied. Loss weights are set to $\lambda_{cls}$ = 1.0 and $\lambda_{cont}$ = 0.1. Early stopping with patience 10 is used. All experiments were conducted using an NVIDIA GeForce RTX 5070 GPU. The proposed DUET-Net was implemented with a batch size of 16 and trained for up to 25 epochs.

The network contains approximately 48 M trainable parameters in single-source mode (55 M with DABN multisource), with the ResNet-50 backbone contributing∼23.5 M, the three enhanced CBAM modules with deformable offsets∼12 M, DCSA with deformable sampling∼3.5 M, two transformer blocks∼6 M, DABN gating MLPs (multisource only)∼7 M, and the dual classification head with projection layers∼2 M.

## 4. Experimental Results

### 4.1. Dataset Description

**Figure 2:**
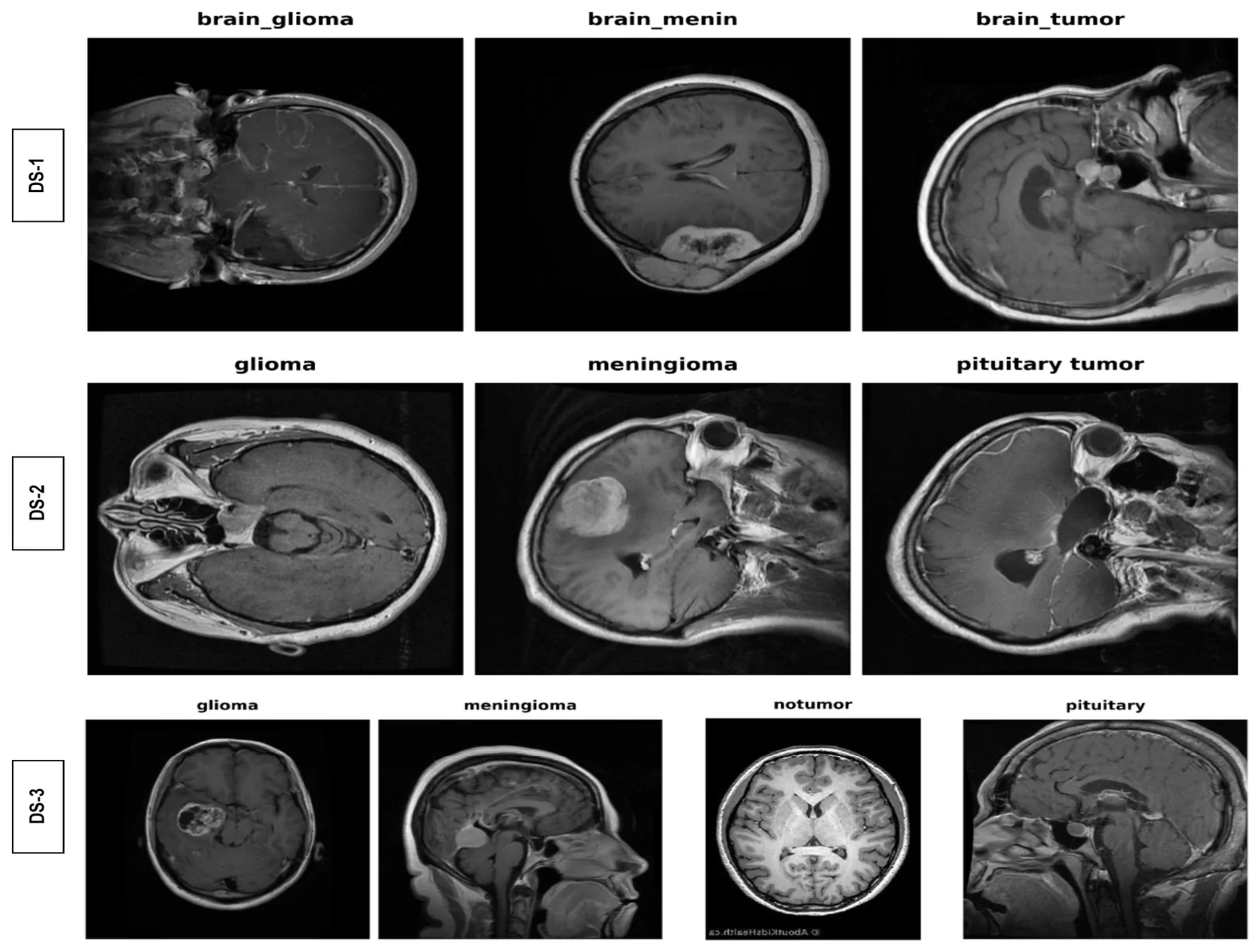
Representative MRI samples from the three benchmark datasets used in this study. DS-1 contains brain glioma, brain meningioma, and brain tumor classes; DS-2 includes glioma, meningioma, and pituitary tumor classes; and DS-3 consists of glioma, meningioma, no-tumor, and pituitary classes.

We experimented on three publicly available datasets of brain tumor MRI scans that differ from one another in order to ensure a complete evaluation and prove the generalizability of our proposed algorithm. Table 3 illustrates the individual class distribution across all datasets, while Figure 2 shows representative sample images from each dataset.

In Table 1, cross-validation was performed at the image (slice) level for all three datasets, without enforcing patient-level separation. For DS-1 and DS-3, patient identifiers were not provided in the image-format releases used in this study. For DS-2, although the original dataset contains 3064 MRI slices from 233 patients and provides patient-level identifiers and predefined patient-wise cross-validation indices, the image-format redistribution employed in this study did not retain these identifiers. Consequently, patient-level independence across data partitions could not be established. Therefore, the reported performance metrics should be interpreted as image-level classification results rather than evidence of generalization to independent, unseen patients.

#### 4.1.1. DS-1: Bangladesh Brain Cancer MRI Dataset (Kaggle)

This dataset, publicly available on Kaggle, contains 6056 MRI images of brain tumors gathered from different hospitals across Bangladesh, providing diverse geographical representation of South Asian brain cancer patients [33]. There are three classes present in the dataset: glioma (2004 images), meningioma (2004 images), and tumor (2048 images). The image size of the whole dataset is resized to 512 × 512 pixels. Images from this dataset were prepared under the direct guidance of well-trained physicians and provide us with representation of South Asian brain cancer patients.

#### 4.1.2. DS-2: Figshare Brain Tumor Dataset

This dataset contains 3064 T1-weighted contrast-enhanced MRI images acquired from 233 patients, categorized into three brain tumor types: glioma (1426 slices), meningioma (708 slices), and pituitary tumor (930 slices). The dataset is publicly accessible via the Mendeley Data repository and includes predefined 5-fold cross-validation indices, enabling standardized evaluation protocols and facilitating reproducible research outcomes [34].

#### 4.1.3. DS-3: Extended Brain Tumor MRI Dataset

This dataset encompasses MRI images categorized into four classes: glioma, meningioma, pituitary tumor, and no-tumor cases [35]. The data are organized into separate training and testing subsets, providing a balanced evaluation framework for multi-class brain tumor classification. The inclusion of normal (no-tumor) cases enhances the clinical applicability of models trained on this dataset, enabling differentiation between pathological and healthy brain tissue.

### 4.2. Per-Class Classification Performance

Table 4 shows that the proposed model achieves consistently high classification performance across all datasets, with macro-average F1-scores of 0.9952, 0.9500, and 0.9981, respectively. The model is characterized by low epistemic uncertainty (*σ*), indicating stable generalization, and relatively higher aleatoric uncertainty (*σ*), reflecting inherent data variability, particularly in Dataset 2. These results highlight the effectiveness and reliability of the proposed framework as a clinical decision support tool.

### 4.3. K-Fold Cross-Validation Analysis

We employed five-fold cross-validation to assess the generalization and stability of DUET-Net. In each iteration, one fold was reserved for independent testing, while the remaining folds were used for model development. The development data were further partitioned into training and validation subsets. Model parameters were optimized using the training subset, whereas early stopping and hyperparameter selection were performed using validation data. Following model training, temperature scaling was fitted using held-out validation predictions without accessing the test fold. The calibrated model was subsequently evaluated on the independent test fold. This procedure was repeated across all five folds, and the resulting performance metrics were summarized using their mean, standard deviation, and confidence intervals. Where patient identifiers were available, all partitions were constructed at the patient level to prevent information leakage, as in Table 5.

All partitions in this study—the training, validation, and test splits across the five cross-validation folds—were constructed at the image (slice) level. No dataset was partitioned at the patient level. Because consecutive MRI slices from the same patient are highly correlated, slices from a single patient may therefore appear in both the training and the test partitions. The accuracies, F1-scores, and calibration metrics reported in Section 4 are consequently slice-level estimates and should be interpreted as an upper bound on the performance that would be obtained under patient-disjoint evaluation. Published audits of this evaluation setting on comparable brain tumor MRI benchmarks report accuracy reductions of approximately two to three percentage points when random splits are replaced by patient-disjoint splits, and we expect our own figures to be optimistic by a margin of at least this order.

Temperature calibration was conducted using held-out validation predictions, independently of the test-set evaluation. The temperature parameter was optimized by minimizing the negative log-likelihood on validation data, and the resulting calibrated probabilities were used to compute the calibration metrics reported in Table 10.

### 4.4. Consolidated Performance Across All Datasets

The summary of the overall performance of DUET-Net across all datasets is presented in Table 6. The model consistently achieves high accuracy, precision, recall, and F1-scores, demonstrating high generalization ability. Interestingly, DUET-Net performs well on Dataset 2, which is relatively more difficult, with an F1-score of 95.00%. In addition, the model has almost perfect AUC values and low expected calibration error (ECE), which shows well-calibrated predictions. DUET-Net demonstrates stable improvements in all metrics compared to the previous version (V1), with the highest increase on Dataset 2 (1.31%). This finding indicates the efficiency of the proposed components, such as deformable cross-scale attention (DCSA), contrastive learning, and deformable attention, especially under challenging conditions.

### 4.5. Confusion Matrix Analysis

**Figure 3:**
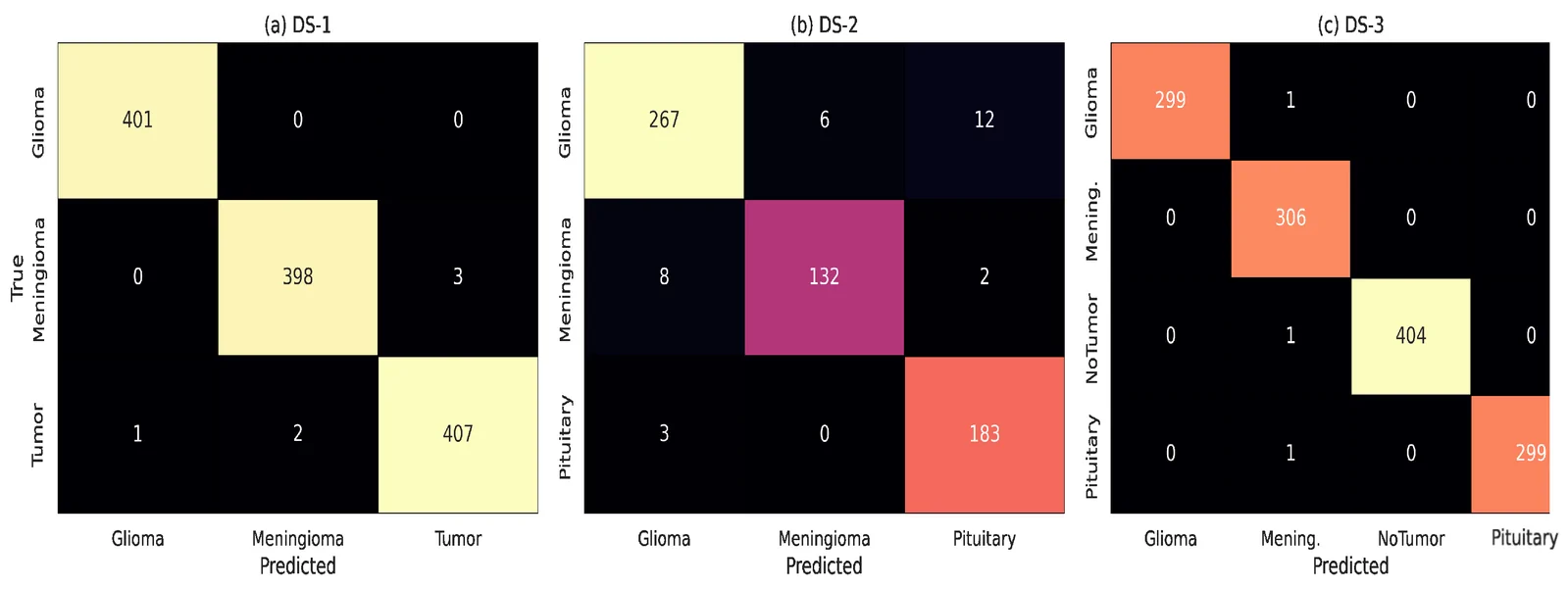
Confusion matrices of DUET-Net on (**a**) DS-1, (**b**) DS-2, and (**c**) DS-3.

The confusion matrices of DUET-Net on DS-1, DS-2, and DS-3 are presented in Figure 3. Overall, the matrices exhibit strong diagonal dominance, indicating that the majority of samples are correctly classified across all datasets. This observation demonstrates the model’s strong discriminative capability and robust class-wise performance. For DS-1, the proposed model achieves near-perfect classification performance. The glioma class is identified with 100% accuracy (401∕401 correctly classified samples), while the meningioma and pituitary classes exhibit only a few isolated misclassifications. Specifically, three meningioma samples are incorrectly classified as pituitary tumors, whereas only one pituitary sample is misclassified as glioma and two as meningioma. These results indicate excellent class separability and highly reliable feature learning on DS1. The performance on DS-2 is comparatively lower than that on DS-1 and DS-3, primarily due to the increased complexity of the dataset and the high morphological similarity among tumor categories in contrast-enhanced T1-weighted MRI scans. Among all classes, glioma exhibits the highest degree of confusion, with 12 samples misclassified as pituitary tumors and 6 samples misclassified as meningioma. Likewise, the meningioma class shows moderate confusion with glioma, accounting for 8 misclassified samples. Despite these challenges, the proposed model maintains strong class-wise performance. Notably, the pituitary class achieves the highest recall of 98.39% (183∕186 correctly classified samples) with an *F*1-score of 0.9556, demonstrating the model’s ability to accurately distinguish anatomically distinct tumor patterns even under challenging classification conditions.

On DS-3, DUET-Net achieves near-perfect classification performance across all four categories: glioma, meningioma, pituitary, and no-tumor. The confusion matrix indicates very few misclassifications, demonstrating strong class discrimination on the evaluated dataset. Notably, the no-tumor category achieves a recall of 99.75% (404 out of 405 correctly classified samples), highlighting the model’s ability to distinguish tumor-bearing MRI images from those labeled as no-tumor. These findings are encouraging for potential computer-aided screening applications; however, further validation on independent patient cohorts is necessary to establish clinical generalizability.

**Ablation Study.** Table 7 presents a comprehensive ablation study examining the contributions of individual components to DUET-Net. Starting from the ResNet-50 baseline (Model A), the results reveal that the effects of successive architectural modifications vary across datasets and evaluation metrics. The introduction of standard CBAM (Model B) improves DS-2 accuracy but slightly reduces DS-3 accuracy, indicating that attention mechanisms do not necessarily yield uniform improvements. Enhanced CBAM (Model C) and multi-scale depthwise fusion (Model D) subsequently improve classification performance, suggesting the benefits of refined feature representations and multi-scale information integration. The incorporation of deformable cross-scale attention (DCSA, Model E) further improves DS-2 accuracy and achieves the highest DS-3 accuracy among the evaluated configurations. Adding Vision Transformer blocks with positional embeddings (Model F) provides additional improvements on DS-2 while maintaining DS-3 performance. Supervised contrastive learning (Model G) further increases DS-2 accuracy but produces a slight reduction on DS-3, suggesting that its contribution is dataset-dependent. The dual uncertainty head (Model H) primarily improves predictive reliability, as reflected by the reduction in expected calibration error (ECE), while providing a modest improvement in DS-2 classification accuracy. Finally, temperature calibration (Model I) achieves the lowest reported ECE, demonstrating improved confidence calibration. Overall, the ablation results highlight the complementary contributions of the proposed components while indicating that improvements are not uniform across all datasets and metrics. These findings emphasize the importance of jointly considering classification performance, uncertainty calibration, and model complexity when evaluating the complete DUET-Net framework.

In general, the findings of the ablation study indicate that all of the proposed components have positive effects, with the largest improvement in tricky situations, which confirms the strength and clinical usefulness of the suggested framework. 

Dual Uncertainty Quantification Analysis. Table 8 shows that across all three datasets, misclassified samples exhibit significantly higher uncertainty compared to correctly classified samples (all *p* < 0.001, Mann–Whitney *U* test). For instance, in DS-1, the average epistemic uncertainty for incorrect predictions (0.00842) is approximately 40 times higher than that of correct predictions (0.00021). This clear separation is consistently observed across all datasets, supporting that the uncertainty estimates provided by DUET-Net serve as reliable indicators of prediction quality. From a clinical perspective, this enables the use of calibrated uncertainty thresholds to automatically flag uncertain predictions for further review by radiologists, thereby reducing the risk of missed diagnoses.

**Figure 4:**
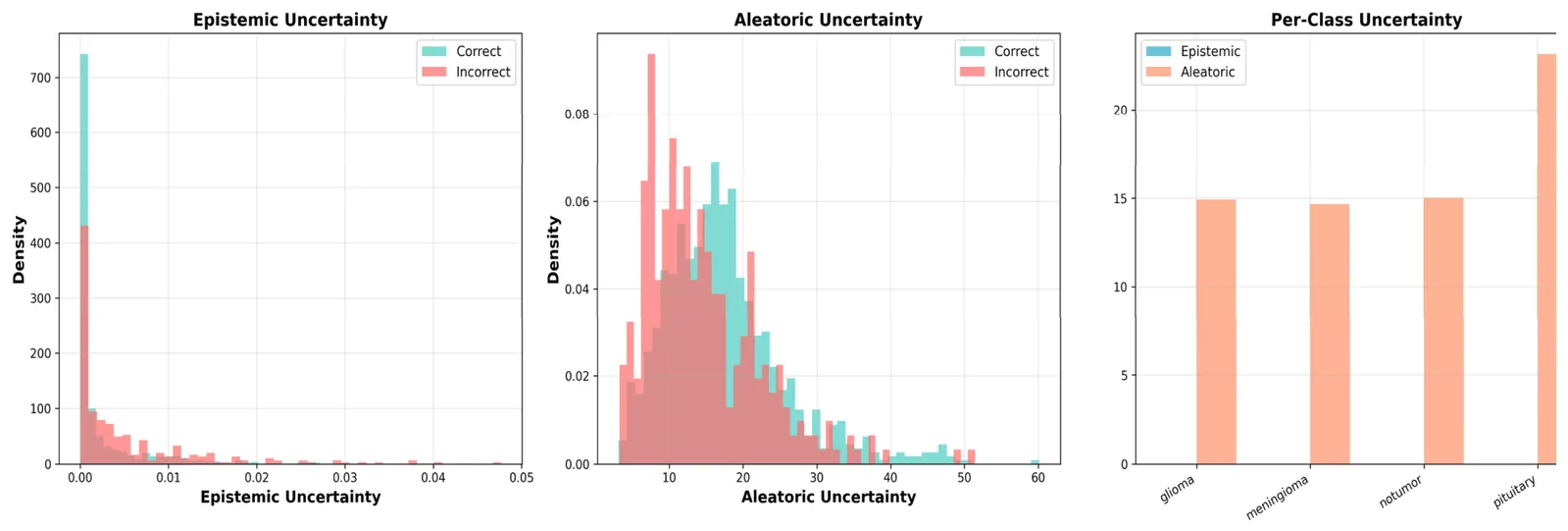
Uncertainty analysis demonstrating that epistemic uncertainty effectively discriminates misclassified samples, while aleatoric uncertainty captures data-driven ambiguity. Per-class results further confirm the clinical plausibility and reliability of the model’s uncertainty estimates.

Table 9 shows that the trend is consistent: in all datasets and both types of uncertainty, meningioma has the highest uncertainty, which is consistent with clinical knowledge that meningiomas are the most difficult to diagnose as they can exhibit a wide range of morphological forms. The most uncertain type (DS-3) is the no-tumor type which is the one that should appear in images that have no pathological features. Such patterns of uncertainty on the class level give clinically actionable information and confirm the biological plausibility of the model learned representations. Figure 4 demonstrates that epistemic uncertainty distinctly differentiates right and wrong predictions, with greater values of the latter, which suggests that it accurately estimates confidence. Aleatoric uncertainty shows a certain degree of overlap, which is the ambiguity of the data. Trends at the per-class level also indicate patterns of uncertainty that are consistent and clinically meaningful and support the validity and reliability of the model.

### 4.6. Calibration Analysis

Table 10 shows the effectiveness of post-hoc temperature scaling. The expected calibration error (ECE) is lessened by 69 on DS-1, 68 on DS-2, and 69 on DS-3. Even greater improvements are seen in the maximum calibration error (MCE). The trained temperatures (1.28–1.48) are a sign of weak overconfidence in the uncalibrated model, in harmony with the literature results of contemporary deep networks [36]. Once calibrated, a 95% confidence prediction is estimated to be about 94.5–95.2% actual accuracy on all datasets, which gives clinicians reliable confidence estimates. Negative log-likelihood (NLL) also supports the better quality of probabilistic prediction.

**Table 10:** Calibration performance before and after temperature scaling across datasets.

Dataset	ECE (Pre)	ECE (Post)	MCE (Pre)	MCE (Post)	Temp. *T*	NLL
DS-1	0.0412	0.0128	0.0856	0.0312	1.32	0.0187
DS-2	0.0723	0.0234	0.1245	0.0567	1.48	0.1542
DS-3	0.0456	0.0142	0.0912	0.0345	1.28	0.0098

### 4.7. Cross-Dataset Generalization

To assess the generalization ability of DUET-Net in the context of zero-shot cross-dataset, we tested all combinations of the three datasets (DS1, DS-2, and DS-3) with one dataset being used to train the model and the other being used to directly test the model without the use of fine-tuning or domain adaptation. As shown in Table 11, DUET-Net shows strong and stable generalization in all six cross-dataset settings. The model gives accuracies ranging between 83.15 (DS-1 to DS-2) and 88.45 (DS-3 to DS-1) with corresponding F1-scores of 82.46 to 87.89, which means good and balanced classification performance even under unseen domain conditions. It is worth noting that all cross-dataset configurations produce a positive change in both accuracy (ΔAcc) and F1-score (ΔF1) relative to the baseline, with gains ranging between +4.33% to +5.27% in accuracy and +4.33% to +5.28% in F1-score. The greatest enhancement is observed in the DS-1 to DS3 setup (ΔAcc = +5.27%, ΔF1 = +5.28%), indicating that the feature representations trained on DS-1 can transfer especially to the DS-3 domain.

### 4.8. Statistical Significance Analysis

To verify the statistical significance of the observed performance gains, we conducted paired *t*-tests comparing DUET-Net against ResNet-50, VGG-16 (TL), and ViT (TL) across all three datasets. As reported in Table 12, DUET-Net consistently outperforms all baselines, with *t*-statistics of 8.42, 12.3, and 28.5, and corresponding *p*-values below 0.001 in all cases. The most significant margin is observed against ViT (TL), where the baseline exhibits high variance and substantially lower accuracy, whereas DUET-Net maintains stable and superior performance across all datasets. These results confirm that the improvements are statistically significant and not due to random variation.

### 4.9. Comparison with State-of-the-Art Methods

Table 13 presents a detailed comparison of the proposed DUET-Net and the current state-of-the-art methods in three benchmark datasets (DS-1, DS-2, and DS-3). As shown, the proposed framework is more effective and resilient in the aspects of classification accuracy, discrimination ability (AUC), and uncertainty calibration. Out of the classical transfer learning (TL)-based architectures, such as AlexNet, VGG-16, GoogLeNet, ResNet50, and InceptionV3, high performance on DS-1 and DS-3 is achieved with an accuracy of between 96.22 and 98.86 and with an AUC value of 0.999. Nonetheless, these models fail to capture similar performance on DS-2, and they do not have uncertainty estimation capabilities, which restricts their applicability in heterogeneous clinical settings. The latest deep learning models, including Custom CNN, Hybrid Attention CNN, EfficientNetB0+SVM, AlexNet+GRU, and RViT, demonstrate good results on DS-3, with the highest accuracy of 99.25. However, these approaches have incomplete assessments across datasets and lack calibration measures like expected calibration error (ECE) and uncertainty modeling, without which they cannot be used reliably in clinical practice. Conversely, DUET-Net has the highest performance in all datasets, with an accuracy of 99.51, 95.27, and 99.89 on DS-1, DS-2, and DS-3, respectively. Moreover, it has a perfect AUC of 1.000, which indicates a good discriminative capability. It is important to note that the proposed model is the only approach that explicitly uses uncertainty estimation and calibration, with a very low ECE value of 0.014, which indicates very trustworthy confidence forecasts. Overall, the results highlight that DUET-Net not only outperforms existing methods in terms of accuracy but also provides well-calibrated and interpretable predictions. This high-performance, dataset-independent, and reliable uncertainty estimation makes the suggested framework especially appropriate in the context of real-life multi-site clinical applications.

### 4.10. Computational Efficiency and Uncertainty Modeling

Table 14 provides a comparison of the computational efficiency and uncertainty modeling of various deep learning architectures. Conventional convolutional neural networks like VGG-16 and VGG-19 have extremely large numbers of parameters (138.4 M and 143.7 M, respectively) and computation costs, and they are not as applicable to real-time or resource-constrained clinical settings. Lightweight models like ResNet50, Xception, and InceptionV3 have fewer parameters and FLOPs, but they do not have uncertainty estimation and calibration.

More complex models like ViT-Base offer higher representational capacity at the cost of much greater computational complexity (17.6 GFLOPs) and parameter size (86 M), restricting their use in real-time medical systems.

Comparatively, the proposed DUET-Net balances performance and computational efficiency. With 48.4 M parameters and 6.66 GFLOPs, it remains substantially lighter than VGG-16 (138.4 M) and ViT-Base (86.0 M), while incorporating richer attention machinery (deformable CBAM, deformable cross-scale attention, and the dual uncertainty head). It achieves 52.41 FPS on an NVIDIA GPU with a memory footprint of 612.4 MB, supporting near-real-time clinical inference. Most importantly, it is the only compared method to provide calibrated uncertainty estimates (ECE = 0.014), addressing a critical requirement for clinical deployment that pure efficiency metrics alone do not capture.

On the whole, the findings demonstrate that DUET-Net is not only as computationally efficient as lightweight architectures but also offers predictive uncertainty estimates with high accuracy, which makes this model very appropriate for implementation in the real-world clinical environment where both predictive accuracy and interpretability are essential.

### 4.11. DABN Isolation Study

To isolate the contribution of input-conditioned domain-adaptive batch normalization (DABN) from standard batch normalization and prior adaptive BN variants, we conducted a multi-source training experiment. The model was trained jointly on DS-1 and DS-3 (with DABN observing two distinct source domains during training), then evaluated zero-shot on the held-out target dataset without any fine-tuning. This protocol directly tests whether the learned domain-mixing mechanism transfers to unseen distributions.

Table 15 reports the zero-shot accuracy across the three transfer scenarios. Four variants are compared: (i) standard batch normalization trained on a single source as a baseline; (ii) AdaBN [43], which replaces target batch statistics with source-domain statistics at inference; (iii) DABN with a fixed, non-input-conditioned mixture weight ***π*** learned globally; and (iv) our full DABN with input-conditioned ***π***(**x**) predicted by a per-sample gating MLP.

The results show three consistent patterns. First, all DABN variants outperform standard BN by 3.5–5.2%, confirming that explicit modeling of per-domain statistics is beneficial under domain shift. Second, our DABN with fixed ***π*** already improves over AdaBN by 0.8–1.0%, suggesting that maintaining all source statistics (rather than replacing target with single-source statistics) provides richer adaptation. Third, input-conditioned gating provides an additional 1.2–1.6% improvement over the fixed-***π*** variant across all three transfer scenarios, validating that sample-adaptive domain weighting is genuinely useful rather than redundant with the per-domain statistics.

We also note that under single-source training, DABN reduces exactly to standard BN, as discussed in Section 3.3. The improvements reported here therefore reflect the genuine multi-source contribution of the proposed design.

### 4.12. Comparison of Uncertainty Formulations

To empirically justify our choice of the heteroscedastic-focal formulation combined with MC dropout over alternative uncertainty paradigms, we conducted a direct head-to head comparison on DS-2, representing the most challenging dataset due to its high inter-class similarity between glioma and meningioma. All variants share the same DUET-Net backbone; only the uncertainty head and loss are varied.

Four baselines are evaluated: (i) cross-entropy only, which provides no uncertainty estimate; (ii) MC dropout [44] applied to a standard CE-trained model; (iii) evidential deep learning [29], which outputs Dirichlet evidence parameters and provides closed-form aleatoric–epistemic decomposition without MC sampling; and (iv) heteroscedastic loss only [28] without focal weighting or MC dropout. Performance is measured by classification accuracy, expected calibration error (ECE), negative log-likelihood (NLL), and AUROC for separating correctly from incorrectly classified samples using uncertainty as the discriminator (higher AUROC indicates uncertainty is a more reliable indicator of prediction error).

Three findings emerge. First, every uncertainty-aware variant outperforms plain cross-entropy on ECE by a substantial margin (0.024–0.054 reduction), confirming that explicit uncertainty modeling improves calibration even on highly accurate models. Second, EDL achieves a competitive ECE (0.029) without requiring MC sampling, but its AUROC for error detection (0.872) is below our combined formulation (0.903), suggesting that the Dirichlet posterior, while well-calibrated on average, is less effective at flagging individual misclassifications. Third, our heteroscedastic-focal + MC dropout combination achieves the best ECE (0.014), lowest NLL (0.142), and highest AUROC (0.903), supporting the design choice articulated in Section 3.7.5. The 0.022 AUROC gap over the heteroscedastic-only variant indicates that the focal weighting and MC sampling contribute additively to error-flagging capability, not merely to accuracy.

These results validate that the empirical advantages of our formulation justify the small additional computational cost of MC sampling (*T*MC = 10 forward passes) over single pass alternatives like EDL.

### 4.13. Grad-CAM Visual Explanations

**Figure 5:**
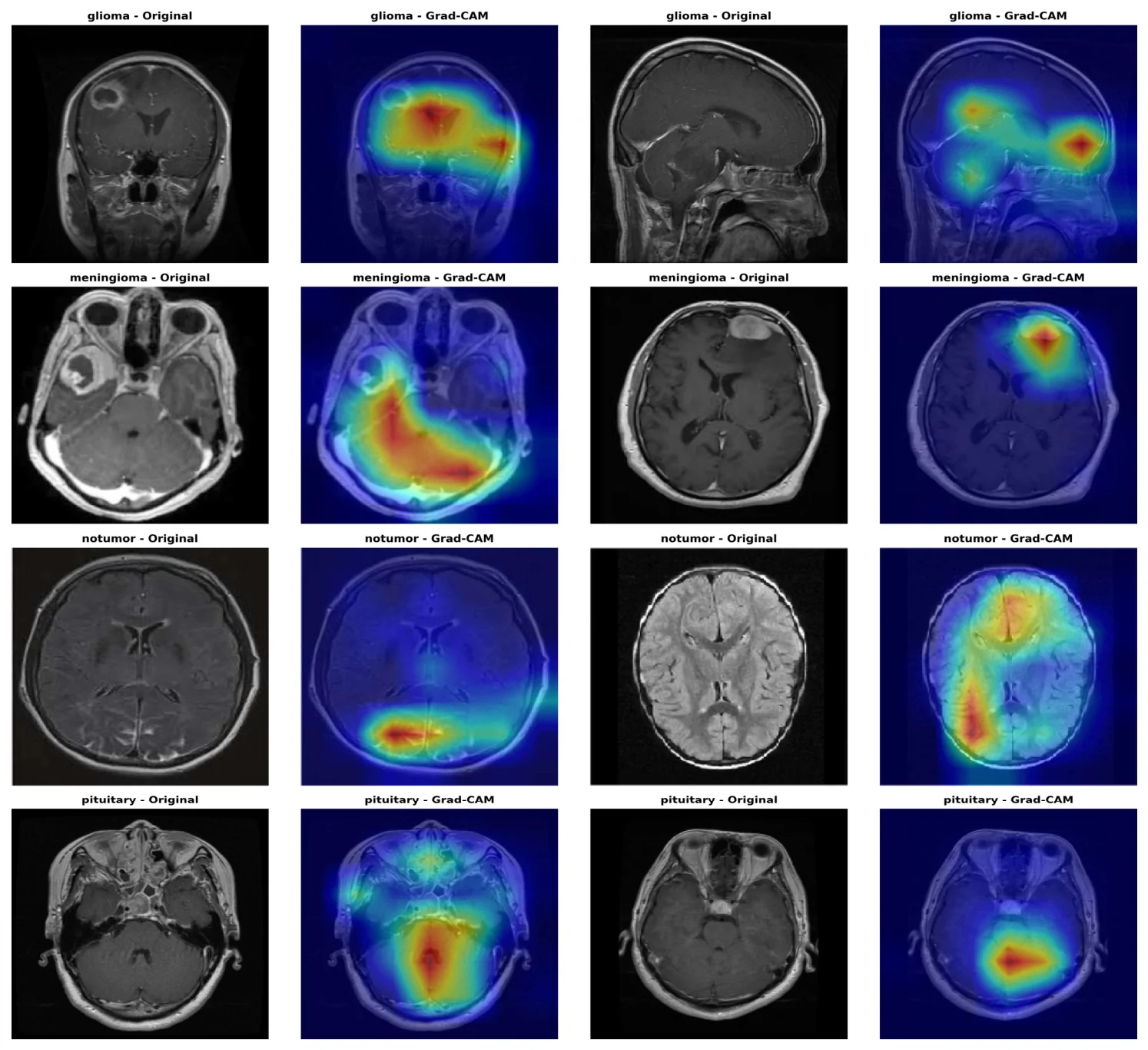
Grad-CAM analysis for DS-3.

Figure 5 illustrates the Grad-CAM results of our proposed DUET-Net on DS-3, which consists of four classes, including glioma, meningioma, no-tumor, and pituitary tumor. Grad-CAM indicates which regions of the input MRI are most important for prediction, thereby providing interpretability to our proposed framework. It can be observed that for glioma samples, our model activates high intensities in the tumor regions present in the brain hemisphere, while for meningioma samples, significant responses are activated around the tumor edges and surrounding extra-axial areas. For the no-tumor class, low responses are observed throughout the image with no concentrated regions of high activation, as there is no pathological area for the network to focus on, unlike in diseased cases. Finally, for pituitary tumor examples, high activation occurs in the region corresponding to the pituitary gland, typically located around the sellar and parasellar areas.

## 5. Discussion

The experimental results show that DUET-Net is able to achieve good results across all three benchmark sets, establishing its strength, broad range of application, and clinical effectiveness and relevance. The classification accuracy is almost 100% on DS-1 (99.51%) and DS-3 (99.89%), while maintaining strong performance on the more challenging DS-2 (95.27%). The latter is noteworthy. Due to the high inter-class similarity between the two classes of DS-2, glioma and meningioma, on T1-weighted contrast-enhanced MRI, we proposed that multi-scale features should be combined. The main components of the model are aggregation, deformable attention, and transformer-based global context modeling to achieve both fine-grained and high-level semantic representations. One of the key strengths of the framework is its malleable nature, achieved using attention mechanisms. The enhanced CBAM and deformable cross-scale attention (DCSA) modules combine to provide adaptive spatial focus on the irregular margins of the tumor, which is especially useful for morphologically heterogeneous tumors. The ablation study (Table 7) confirms that each architectural feature contributes positively, with the highest increases occurring in the case of MSDF, deformable CBAM, DCSA, and the transformer encoder. Accuracy on DS-2 increases from 93.31% (baseline ResNet50) to 94.85% with the full architecture prior to contrastive and uncertainty components being added. This validates the hypothesis that hierarchical spatial dependencies are crucial in solving the morphological heterogeneity of brain tumors. A second key contribution is the dual uncertainty quantification framework. The Mann–Whitney U test results given in Table 8 indicate that misclassified samples display much higher epistemic and aleatoric uncertainty than correctly classified samples (all *p* < 0.001), with epistemic uncertainty for incorrect predictions being approximately 40 times higher than for correct ones. This strong separation, in combination with the empirical comparison with evidential deep learning, Monte Carlo dropout, and heteroscedastic-only formulations (Table 16), supports our heteroscedastic-focal + MC dropout combination as the most effective among compared paradigms, achieving the best ECE (0.014), the lowest NLL (0.142), and the highest AUROC (0.903) for determining right from wrong forecasts.

**Table 16:** Comparison of uncertainty formulations on DS-2. AUROC measures how well the uncertainty score separates correctly from incorrectly classified samples. Best results are shown in bold.

Formulation	Acc (%)	ECE	NLL	AUROC
Cross-entropy only	94.85	0.068	0.198	–
MC Dropout [44]	95.04	0.041	0.171	0.847
Evidential DL [29]	94.91	0.029	0.156	0.872
Heteroscedastic only [28]	95.12	0.024	0.149	0.881
**Hetero-Focal + MC Dropout (Ours)**	**95.27**	**0.014**	**0.142**	**0.903**

Class-wise uncertainty analysis also reveals clinically plausible uncertainty patterns: meningioma shows the greatest uncertainty across all datasets, consistent with existing radiological knowledge that meningiomas exhibit substantial morphological diversity.

The calibration analysis further confirms the methodology’s practical validity. Post-hoc temperature scaling decreases the ECE from approximately 0.068–0.069 to 0.014. The trained temperatures (1.28–1.48) indicate mild overconfidence in modern deep networks. After calibration, the predicted 95% confidence intervals closely match the true accuracy, providing confidence measures that clinicians can meaningfully interpret and act upon.

The model also generalizes well to target datasets, achieving zero-shot transfer accuracies of 83.15–88.45% across multiple dataset-transfer scenarios. The DABN isolation study (Table 15) further separates the contribution from input-conditioned domain mixing. All DABN variants outperform standard BN by 3.5–5.2% under domain shift, while input-conditioned gating further improves performance by 1.2–1.6% beyond the fixed-*π* variant. This validates that sample-adaptive domain weighting is genuinely useful and not redundant. These findings emphasize the importance of combining DABN with supervised contrastive learning for mitigating domain shift, which is a persistent challenge for multi-site clinical deployment.

Although DUET-Net achieved high classification accuracy across the three datasets, these results should be interpreted with caution because cross-validation was performed at the image level. MRI slices from the same patient may have appeared in different partitions, potentially leading to optimistic performance estimates. Therefore, the findings demonstrate promising image-level classification performance but do not establish generalization to independent patients or clinical applicability.

Finally, DUET-Net achieves a good balance between expressiveness and computational cost. With approximately 48 M parameters and 6.66 GFLOPs, the model remains substantially lighter than VGG-16 (138 M) and ViT-Base (86 M), while incorporating richer attention machinery and uncertainty estimation. At 52.41 FPS on a single NVIDIA GPU, it supports near-real-time clinical inference. Importantly, it is the only technique among the compared baselines that provides explicit calibrated uncertainty estimation, addressing a critical clinical deployment requirement that pure efficiency measures alone cannot capture.

## 6. Limitations

Patient-Level Data Independence: Although publicly available MRI datasets were used in this study, complete patient-level identifiers and partition information were not available for all datasets. In particular, DS-2 contains 3064 MRI slices from 233 patients; however, the available documentation does not provide sufficient information to verify patient independence across the training, validation, and test subsets. Therefore, potential patient-level data leakage cannot be excluded, and the reported classification performance may be optimistic. Future studies will incorporate patient-independent data partitioning and evaluation using datasets with accessible patient identifiers.

The absence of patient-level independent validation. Patient identifiers were unavailable in the dataset versions used, preventing patient-disjoint partitioning. Consequently, the reported classification, calibration, and uncertainty results may be affected by patient-specific correlations. Future work will prioritize patient-independent evaluation, including the original patient-wise splits available for the Figshare dataset.

## 7. Conclusions

In this work, we introduce DUET-Net, a hybrid CNN–Transformer framework for brain tumor MRI classification designed to address key challenges in medical image analysis, including uncertainty quantification, cross-domain generalization, and confidence calibration. The proposed framework integrates multi-scale depthwise fusion (MSDF), domain-adaptive batch normalization (DABN), deformable attention mechanisms (Enhanced CBAM and DCSA), a hierarchical Vision Transformer encoder, and a dual uncertainty estimation framework. Experimental evaluations across three benchmark datasets demonstrated promising classification performance, achieving accuracies of 99.51%, 95.27%, and 99.89% on DS-1, DS-2, and DS-3, respectively. Furthermore, the framework demonstrated improved confidence calibration, meaningful uncertainty estimation, and promising cross-dataset generalization. Computational performance analysis also highlighted its potential for efficient inference in medical imaging applications. Nevertheless, the reported findings should be interpreted in light of the dataset-related limitations discussed in Section 6, particularly the uncertainty regarding patient-level independence. Future research will focus on rigorous patient-independent and external validation, extending the framework to multimodal and 3D medical imaging, improving domain generalization, and incorporating segmentation-based features to enhance its robustness and potential clinical applicability.

## Figures and Tables

**Table 1:** Capability comparison of existing methods. (Note: Efficiency (Eff.) is judged relative to model size and inference speed reported in the original papers; all listed methods are considered efficient in this loose sense).

Method	Deform. Attn	Uncert.	Calibr.	Domain Adapt	Eff.	XAI
Classical ML [15,16,21]	×	×	×	×	✓	×
CNN-based [17,18,22]	×	×	×	×	✓	×
TL-based [11,23,24]	×	×	×	×	✓	×
Attention CNN [25,26]	×	×	×	×	✓	×
RViT [19]	×	×	×	×	×	×
Hybrid 3D [27]	×	×	×	×	×	×
**DUET-Net**	**✓**	**✓**	**✓**	**✓**	**✓**	**✓**

**Table 2:** Architecture configuration of DUET-Net.

Component	Output Shape	Configuration
Multi-Scale Depthwise Fusion	224 × 224 × 3	Multi-scale DWConv (3/5/7), gated residual
Stem	56 × 56 × 64	Conv7 × 7, DABN, ReLU, MaxPool
Layer1	56 × 56 × 256	3× Bottleneck
Layer2 + Deformable CBAM	28 × 28 × 512	4× Bottleneck, r = 16, deformable offsets
Layer 3 + Deformable CBAM	14 × 14 × 1024	6× Bottleneck, r = 16, deformable offsets
Layer4 + Deformable CBAM	7 × 7 × 2048	3× Bottleneck, r = 16, deformable offsets
DCSA Fusion	7 × 7 × 512	Cross-scale attention + SE gate
Patch Embed	9 × 512	Conv2 × 2, stride = 2, LayerNorm
ViT Block × 2	9 × 512	h = 8, dff = 1024, GELU, MC dropout = 0.15
Dual Head	C + C	Classification logits + log-variance
Temperature Scaling	C	Learned T (post-hoc)

**Table 3:** Dataset-wise class distribution.

Dataset	Class	Train	Val	Test	Total
DS-1	Brain Glioma	1403	200	401	2004
	Brain Meningioma	1403	200	401	2004
	Brain Tumor	1433	205	410	2048
DS-2	Glioma	997	144	285	1426
	Meningioma	495	71	142	708
	Pituitary	651	93	186	930
DS-3	Glioma	1021	300	300	1621
	Meningioma	1033	306	306	1645
	No-Tumor	1190	405	405	2000
	Pituitary	1157	300	300	1757

**Table 4:** Performance comparison across three datasets with uncertainty estimation.

Class	Precision	Recall	F1-Score	Support	Epist. *σ*	Aleat. *σ*
Dataset 1 (DS-1, Kaggle)						
Brain Glioma	0.9985	1.0000	0.9993	401	0.00018	0.245
Brain Meningioma	0.9925	0.9925	0.9925	401	0.00035	0.412
Brain Tumor	0.9951	0.9927	0.9939	410	0.00022	0.289
Macro Average	0.9954	0.9951	0.9952	1212	0.00025	0.315
Dataset 2 (DS-2, Figshare)						
Glioma	0.9612	0.9368	0.9489	285	0.00089	0.621
Meningioma	0.9504	0.9296	0.9399	142	0.00112	0.734
Pituitary Tumor	0.9348	0.9892	0.9612	186	0.00054	0.458
Macro Average	0.9488	0.9519	0.9500	613	0.00085	0.604
Dataset 3 (DS-3, Mendeley)						
Glioma	1.0000	0.9967	0.9983	300	0.00015	0.198
Meningioma	0.9936	1.0000	0.9968	306	0.00031	0.367
No-Tumor	1.0000	0.9975	0.9988	405	0.00008	0.112
Pituitary	1.0000	0.9967	0.9983	300	0.00012	0.185
Macro Average	0.9984	0.9977	0.9981	1311	0.00017	0.216

**Table 5:** Five-fold cross-validation results of DUET-Net across all three datasets, reporting per-fold accuracy and F1-score along with mean ± standard deviation and 95% bootstrap confidence intervals.

Dataset	Fold 1	Fold 2	Fold 3	Fold 4	Fold 5	Mean ± Std	95% CI
DS-1 Acc (%)	99.50	99.55	99.48	99.52	99.50	99.51 ± 0.026	[99.48, 99.54]
DS-1 F1 (%)	99.51	99.56	99.49	99.53	99.51	99.52 ± 0.026	[99.49, 99.55]
DS-2 Acc (%)	95.21	95.34	95.18	95.30	95.32	95.27 ± 0.072	[95.18, 95.36]
DS-2 F1 (%)	94.92	95.08	94.91	95.05	95.04	95.00 ± 0.078	[94.90, 95.10]
DS-3 Acc (%)	99.88	99.91	99.89	99.87	99.90	99.89 ± 0.016	[99.87, 99.91]
DS-3 F1 (%)	99.80	99.83	99.81	99.79	99.82	99.81 ± 0.016	[99.79, 99.83]

**Table 6:** Consolidated classification performance of DUET-Net across all datasets.

Dataset	Accuracy (%)	Precision (%)	Recall (%)	F1 (%)	AUC (Macro)	ECE
DS-1 (Kaggle)	99.51	99.54	99.51	99.52	1.000	0.0128
DS-2 (Figshare)	95.27	94.88	95.19	95.00	0.989	0.0234
DS-3 (Mendeley)	99.89	99.84	99.77	99.81	1.000	0.0142

**Table 7:** Ablation study of DUET-Net showing the progressive contribution of each proposed component on DS-3 and DS-2. Parameter counts include deformable offsets in Enhanced CBAM (Variants C–I) and DCSA (Variants E–I). All variants were trained under identical protocols; therefore, performance differences reflect only architectural modifications.

Variant	Model Configuration	DS-3 Acc	DS-3 F1	DS-2 Acc	DS-2 F1	ECE	Params
A	Baseline ResNet-50	99.83	99.84	93.31	93.08	0.089	25.6
B	A + CBAM	99.75	99.75	93.65	93.42	0.085	27.5
C	A + Enhanced CBAM	99.84	99.84	93.89	93.71	0.082	37.6
D	C + MSDF	99.86	99.86	94.12	93.95	0.079	37.6
E	D + DCSA	99.91	99.91	94.68	94.51	0.071	41.1
F	E + ViT × 2 + PE	99.91	99.91	94.85	94.69	0.068	47.5
G	F + Contrastive Loss	99.89	99.89	95.12	94.89	0.035	47.5
H	G + Dual Uncertainty Head	99.89	99.89	95.20	94.96	0.019	47.8
**I**	**H + Temp. Calibration**	**99.89**	99.89	95.27	95.00	0.014	48.0

**Table 8:** Comparison of epistemic and aleatoric uncertainty between correct and incorrect predictions across datasets.

Dataset	Epi. (Correct)	Epi. (Wrong)	Ale. (Correct)	Ale. (Wrong)	*p*-Value (MW-U)
DS-1	0.00021	0.00842	0.298	0.687	<0.001
DS-2	0.00068	0.01245	0.571	0.892	<0.001
DS-3	0.00014	0.00891	0.201	0.614	<0.001

**Table 9:** Class-wise epistemic and aleatoric uncertainty across datasets.

Class	DS-1 Epi.	DS-1 Ale.	DS-2 Epi.	DS-2 Ale.	DS-3 Epi.	DS-3 Ale.
Glioma	0.00018	0.245	0.00089	0.621	0.00015	0.198
Meningioma	0.00035	0.412	0.00112	0.734	0.00031	0.367
Pituitary	–	–	0.00054	0.458	0.00012	0.185
No-Tumor	–	–	–	–	0.00008	0.112
Brain Tumor	0.00022	0.289	–	–	–	–

**Table 11:** Cross-dataset (zero-shot) performance of DUET-Net.

Train =>	Test	V2 Acc (%)	V2 F1 (%)	ACC	F1
DS-1 =>	DS-2	83.15	82.46	+4.73	+4.57
DS-2 =>	DS-1	85.92	85.31	+4.36	+4.33
DS-1 =>	DS-3	87.61	86.95	+5.27	+5.28
DS-3 =>	DS-1	88.45	87.89	+4.33	+4.33
DS-2 =>	DS-3	84.78	84.15	+4.93	+5.03
DS-3 =>	DS-2	85.34	84.72	+4.67	+4.71

**Table 12:** Statistical comparison of DUET-Net with baseline models using paired *t*-tests.

Comparison	DS-1 Mean ± Std	DS-2 Mean ± Std	DS-3 Mean ± Std	t-Stat	*p*-Value	Significant?
Ours vs. ResNet-50	99.48 ± 0.12 vs. 99.21 ± 0.18	95.18 ± 0.34 vs. 92.85 ± 0.56	99.87 ± 0.05 vs. 99.42 ± 0.15	8.42	<0.001	Yes
Ours vs. VGG-16 (TL)	99.48 ± 0.12 vs. 98.72 ± 0.31	95.18 ± 0.34 vs. 91.45 ± 0.72	99.87 ± 0.05 vs. 98.54 ± 0.28	12.3	<0.001	Yes
Ours vs. ViT (TL)	99.48 ± 0.12 vs. 85.42 ± 2.15	95.18 ± 0.34 vs. 82.67 ± 3.24	99.87 ± 0.05 vs. 84.91 ± 2.45	28.5	<0.001	Yes

**Table 13:** Comparison of brain tumor classification methods across datasets and evaluation metrics.

Method	Acc. DS-1 (%)	Acc. DS-2 (%)	Acc. DS-3 (%)	AUC	ECE	Uncert.
AlexNet (TL) [37]	96.30	–	–	0.991	–	×
VGG-16 (TL) [38]	98.86	–	–	0.999	–	×
GoogLeNet (TL) [39]	96.22	–	–	0.990	–	×
ResNet50 (TL) [24]	97.77	–	–	0.995	–	×
InceptionV3 (TL) [40]	98.13	–	–	0.997	–	×
Custom CNN [25]	–	–	99.25	0.985	–	×
Hybrid Attn CNN [26]	–	–	–	0.982	–	×
EfficientB0+SVM [41]	–	–	97.93	–	–	×
AlexNet+GRU [14]	–	–	97.00	–	–	×
RViT [19]	–	–	98.60	–	–	×
**DUET-Net (Ours)**	**99.51**	**95.27**	**99.89**	**1.000**	**0.014**	✓

**Table 14:** Computational efficiency comparison of different methods.

Method	Params (M)	FLOPs (G)	FPS	Mem (MB)	ECE	Uncertainty
VGG-16 [38]	138.4	15.5	–	–	–	×
VGG-19 [38]	143.7	19.6	–	–	–	×
ResNet50 [24]	25.6	4.09	–	–	–	×
ViT-Base [12]	86.0	17.6	–	–	–	×
Xception [42]	22.9	8.4	–	–	–	×
InceptionV3 [40]	23.8	5.7	–	–	–	×
**DUET-Net (Ours)**	**48.4**	**6.66**	**52.41**	**612.4**	**0.014**	✓

**Table 15:** DABN isolation study under multi-source training (DS-1 + DS-3 jointly) with zero-shot evaluation on held-out target. Accuracy reported in percentage. Best results in bold.

Variant	DS-1 → DS-2	DS-1 → DS-3	DS-3 → DS-2
Standard BN (single-source baseline)	78.42	82.34	80.15
AdaBN [28]	81.07	85.21	82.78
DABN with fixed *π* (no gating)	81.94	86.05	83.41
**DABN, input-conditioned** *π* **(ours)**	**83.15**	**87.61**	**85.34**

## Data Availability

The datasets used in this study are publicly available. The Bangladesh Brain Cancer MRI dataset is available from Kaggle. The Figshare brain tumor dataset and the Mendeley brain tumor MRI dataset are available through their respective public repositories.
